# Mitochondrial inhibitor sensitizes non-small-cell lung carcinoma cells to TRAIL-induced apoptosis by reactive oxygen species and Bcl-X_L_/p53-mediated amplification mechanisms

**DOI:** 10.1038/cddis.2014.547

**Published:** 2014-12-18

**Authors:** Y-L Shi, S Feng, W Chen, Z-C Hua, J-J Bian, W Yin

**Affiliations:** 1 The State Key Lab of Pharmaceutical Biotechnology, College of Life Sciences, Nanjing University, Nanjing, China; 2 Department of Anesthesiology and Intensive Care Unit, Changhai Hospital, Affiliated Hospital of the Second Military Medical University, Shanghai, China; 3 The State Key Lab of Natural Medicines, China Pharmaceutical University, Nanjing, China

## Abstract

Tumor necrosis factor-related apoptosis-inducing ligand (TRAIL) is a promising agent for anticancer therapy; however, non-small-cell lung carcinoma (NSCLC) cells are relatively TRAIL resistant. Identification of small molecules that can restore NSCLC susceptibility to TRAIL-induced apoptosis is meaningful. We found here that rotenone, as a mitochondrial respiration inhibitor, preferentially increased NSCLC cells sensitivity to TRAIL-mediated apoptosis at subtoxic concentrations, the mechanisms by which were accounted by the upregulation of death receptors and the downregulation of c-FLIP (cellular FLICE-like inhibitory protein). Further analysis revealed that death receptors expression by rotenone was regulated by p53, whereas c-FLIP downregulation was blocked by Bcl-X_L_ overexpression. Rotenone triggered the mitochondria-derived reactive oxygen species (ROS) generation, which subsequently led to Bcl-X_L_ downregulation and PUMA upregulation. As PUMA expression was regulated by p53, the PUMA, Bcl-X_L_ and p53 in rotenone-treated cells form a positive feedback amplification loop to increase the apoptosis sensitivity. Mitochondria-derived ROS, however, promote the formation of this amplification loop. Collectively, we concluded that ROS generation, Bcl-X_L_ and p53-mediated amplification mechanisms had an important role in the sensitization of NSCLC cells to TRAIL-mediated apoptosis by rotenone. The combined TRAIL and rotenone treatment may be appreciated as a useful approach for the therapy of NSCLC that warrants further investigation.

Tumor necrosis factor-related apoptosis-inducing ligand (TRAIL) has emerged as a promising cancer therapeutic because it can selectively induce apoptosis in tumor cells *in vitro*, and most importantly, *in vivo* with little adverse effect on normal cells.^1^ However, a number of cancer cells are resistant to TRAIL, especially highly malignant tumors such as lung cancer.^2, 3^ Lung cancer, especially the non-small-cell lung carcinoma (NSCLC) constitutes a heavy threat to human life. Presently, the morbidity and mortality of NSCLC has markedly increased in the past decade,^4^ which highlights the need for more effective treatment strategies.

TRAIL has been shown to interact with five receptors, including the death receptors 4 and 5 (DR4 and DR5), the decoy receptors DcR1 and DcR2, and osteoprotegerin.^5^ Ligation of TRAIL to DR4 or DR5 allows for the recruitment of Fas-associated protein with death domain (FADD), which leads to the formation of death-inducing signaling complex (DISC) and the subsequent activation of caspase-8/10.^6^ The effector caspase-3 is activated by caspase-8, which cleaves numerous regulatory and structural proteins resulting in cell apoptosis. Caspase-8 can also cleave the Bcl-2 inhibitory BH3-domain protein (Bid), which engages the intrinsic apoptotic pathway by binding to Bcl-2-associated X protein (Bax) and Bcl-2 homologous antagonist killer (BAK). The oligomerization between Bcl-2 and Bax promotes the release of cytochrome c from mitochondria to cytosol, and facilitates the formation of apoptosome and caspase-9 activation.^7^ Like caspase-8, caspase-9 can also activate caspase-3 and initiate cell apoptosis. Besides apoptosis-inducing molecules, several apoptosis-inhibitory proteins also exist and have function even when apoptosis program is initiated. For example, cellular FLICE-like inhibitory protein (c-FLIP) is able to suppress DISC formation and apoptosis induction by sequestering FADD.^8, 9, 10, 11^

Until now, the recognized causes of TRAIL resistance include differential expression of death receptors, constitutively active AKT and NF-*κ*B,^12, 13^ overexpression of c-FLIP and IAPs, mutations in Bax and BAK gene.^2^ Hence, resistance can be overcome by the use of sensitizing agents that modify the deregulated death receptor expression and/or apoptosis signaling pathways in cancer cells.^5^ Many sensitizing agents have been developed in a variety of tumor cell models.^2^ Although the clinical effectiveness of these agents needs further investigation, treatment of TRAIL-resistant tumor cells with sensitizing agents, especially the compounds with low molecular weight, as well as prolonged plasma half-life represents a promising trend for cancer therapy.

Mitochondria emerge as intriguing targets for cancer therapy. Metabolic changes affecting mitochondria function inside cancer cells endow these cells with distinctive properties and survival advantage worthy of drug targeting, mitochondria-targeting drugs offer substantial promise as clinical treatment with minimal side effects.^14, 15, 16^ Rotenone is a potent inhibitor of NADH oxidoreductase in complex I, which demonstrates anti-neoplastic activity on a variety of cancer cells.^17, 18, 19, 20, 21^ However, the neurotoxicity of rotenone limits its potential application in cancer therapy. To avoid it, rotenone was effectively used in combination with other chemotherapeutic drugs to kill cancerous cells.^22^

In our previous investigation, we found that rotenone was able to suppress membrane Na^+^,K^+^-ATPase activity and enhance ouabain-induced cancer cell death.^23^ Given these facts, we wonder whether rotenone may also be used as a sensitizing agent that can restore the susceptibility of NSCLC cells toward TRAIL-induced apoptosis, and increase the antitumor efficacy of TRAIL on NSCLC. To test this hypothesis, we initiated this study.

## Results

### Rotenone sensitizes NSCLC cell lines to TRAIL-induced apoptosis

**Figure 1:**
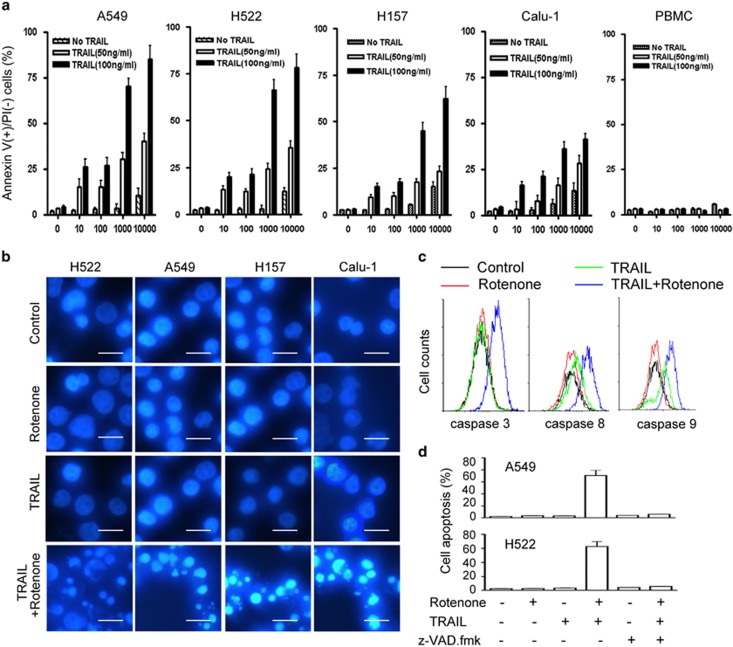
Effects of rotenone and TRAIL on NSCLC and PBMC cell apoptosis. (**a**) A549, H522, H157, Calu-1 and human PBMC cells were treated with rotenone at concentrations ranging from 10 ng/ml to 10 000 nM, or TRAIL at 50, 100 ng/ml, or both for 8 h, after treatment, cells were collected and the occurrence of apoptosis was measured by annexin V/PI double staining method. (**b**) NSCLC cells, including A549, H522, H157 and Calu-1 cells, were treated with rotenone at 1 *μ*M, TRAIL at 100 ng/ml or both for 8 h, after treatment, cells were fixed and stained with Hoechst, then observed under Carl Zeiss fluorescent microscope (Carl Zeiss, Jena, Germany). Scale bars, 20 *μ*M. (**c**) A549 cells were treated with rotenone at 1 *μ*M, TRAIL at 100 ng/ml or both for 8 h, after treatment, cells were collected and stained with FITC-conjugated substrates for caspase-3, -8 and -9. FITC fluorescent intensity within each sample was measured by flow cytometry. The representative overlaid histograms of three independent experiments with similar results are shown. (**d**) A549 or H522 cells were preincubated with 4 *μ*M z-VAD.fmk for 30 min, and further treated with TRAIL (100 ng/ml) and rotenone (1 *μ*M) for 8 h. After treatment, cell apoptosis was assessed by annexin V/PI double staining method. Each experiment was performed at least in triplicate

Four NSCLC cell lines including A549, H522, H157 and Calu-1 were used in this study. As shown in Figure 1a, the apoptosis induced by TRAIL alone at 50 or 100 ng/ml on A549, H522, H157 and Calu-1 cells was non-prevalent, indicating that these NSCLC cell lines are relatively TRAIL resistant. Interestingly, when these cells were treated with TRAIL combined with rotenone, significant increase in cell apoptosis was observed. To examine whether rotenone was also able to sensitize normal cells to TRAIL-mediated apoptosis, peripheral blood mononuclear cell (PBMC) isolated from human blood were used. As a result, rotenone failed to sensitize human PBMC to TRAIL-induced apoptosis, indicating that the sensitizing effect of rotenone is tumor cell specific. Of note, the apoptosis-enhancing effect of rotenone occurred independent of its cytotoxicity, because the minimal dosage required for rotenone to cause toxic effect on NSCLC cell lines was 10 *μ*M, however, rotenone augmented TRAIL-mediated apoptosis when it was used as little as 10 nM.

To further confirm the effect of rotenone, cells were stained with Hoechst and observed under fluorescent microscope (Figure 1b). Consistently, the combined treatment of rotenone with TRAIL caused significant nuclear fragmentation in A549, H522, H157 and Calu-1 cells. Rotenone or TRAIL treatment alone, however, had no significant effect.

Caspases activation is a hallmark of cell apoptosis. In this study, the enzymatic activities of caspases including caspase-3, -8 and -9 were measured by flow cytometry by using FITC-conjugated caspases substrate (Figure 1c). As a result, rotenone used at 1 *μ*M or TRAIL used at 100 ng/ml alone did not cause caspase-3, -8 and -9 activation. The combined treatment, however, significantly increased the enzymatic activities of them. Moreover, A549 or H522 cell apoptosis by TRAIL combined with rotenone was almost completely suppressed in the presence of z-VAD.fmk, a pan-caspase inhibitor (Figure 1d). All of these data indicate that both intrinsic and extrinsic pathways are involved in the sensitizing effect of rotenone on TRAIL-mediated apoptosis in NSCLC.

### Upregulation of death receptors expression is required for rotenone-mediated sensitization to TRAIL-induced apoptosis

**Figure 2:**
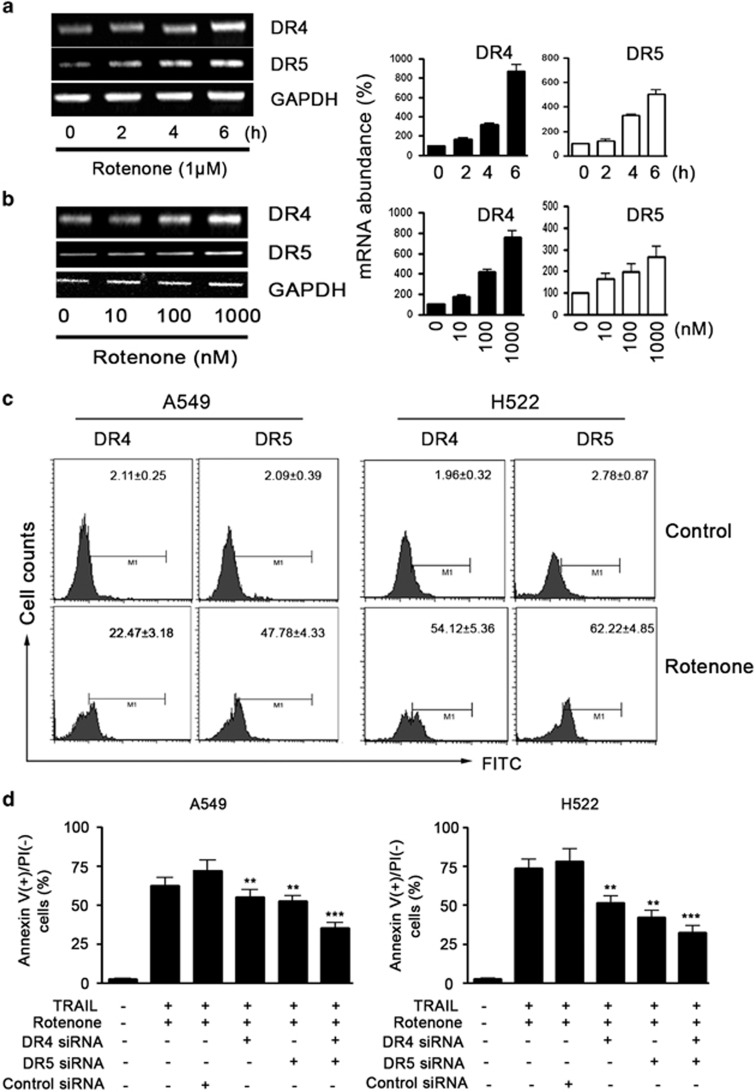
Effect of rotenone on DR4 and DR5 expression levels in NSCLC cells. A549 cells were treated with rotenone at 1 *μ*M for 0, 2, 4 and 6 h, respectively, in **a**, or treated with rotenone at 0, 10, 100 and 1000 ng/ml for 6 h in **b**, after treatment, the total cellular mRNAs were extracted by TRIZOL agent, RT-PCR analysis was performed to measure the DR4 and DR5 mRNA levels. GAPDH was included as a control. The quantitative results of DR4 and DR5 mRNA expression levels are shown on the right panel; the mRNA expression levels of DR4 and DR5 in controls were arbitrarily set at 100%. (**c**) A549 or H522 cells were treated with rotenone at 1 *μ*M for 8 h, after treatment, cells were collected, incubated with DR4 or DR5 monoclonal antibody and FITC-conjugated secondary IgGs. The representative histograms of three independent experiments with similar results are shown. (**d**) A549 or H522 cells were transfected with control siRNA, DR4 or DR5 siRNAs for 24 h, then treated with rotenone (1 *μ*M) and TRAIL (100 ng/ml) for 8 h. After treatment, cells were collected, and cell apoptosis was measured by annexin V/PI double staining. Each experiment was performed in triplicate. **P<0.01, ***P<0.001, as compared with cells transfected with control siRNA

Sensitization to TRAIL-induced apoptosis has been explained in some studies by upregulation of death receptors,^24^ whereas other results show that sensitization can occur without increased TRAIL receptor expression.^25^ As such, we examined TRAIL receptors expression on NSCLC cells after treatment with rotenone. Rotenone increased DR4 and DR5 mRNA levels in A549 cells in a time or concentration-dependent manner (Figures 2a and b), also increased DR4 and DR5 protein expression levels (Supplementary Figure S1). Notably, rotenone failed to increase DR5 mRNA levels in H157 and Calu-1 cells (Supplementary Figure S2). To observe whether the increased DR4 and DR5 mRNA levels finally correlated with the functional molecules, we examined the surface expression levels of DR4 and DR5 by flow cytometry. The results, as shown in Figure 2c demonstrated that the cell surface expression levels of DR4 and DR5 were greatly upregulated by rotenone in either A549 cells or H522 cells.

To analyze whether the upregulation of DR4 and DR5 is a ‘side-effect', or contrarily, necessary for rotenone-mediated sensitization to TRAIL-induced apoptosis, we blocked upregulation of the death receptors by small interfering RNAs (siRNAs) against DR4 and DR5 (Supplementary Figure S3). The results showed that blocking DR4 and DR5 expression alone significantly reduced the rate of cell apoptosis in A549 cells (Figure 2d). However, the highest inhibition of apoptosis was observed when upregulation of both receptors was blocked in parallel, thus showing an additive effect of blocking DR4 and DR5 at the same time. Similar results were also obtained in H522 cells.

### Rotenone-induced p53 activation regulates death receptors upregulation

**Figure 3:**
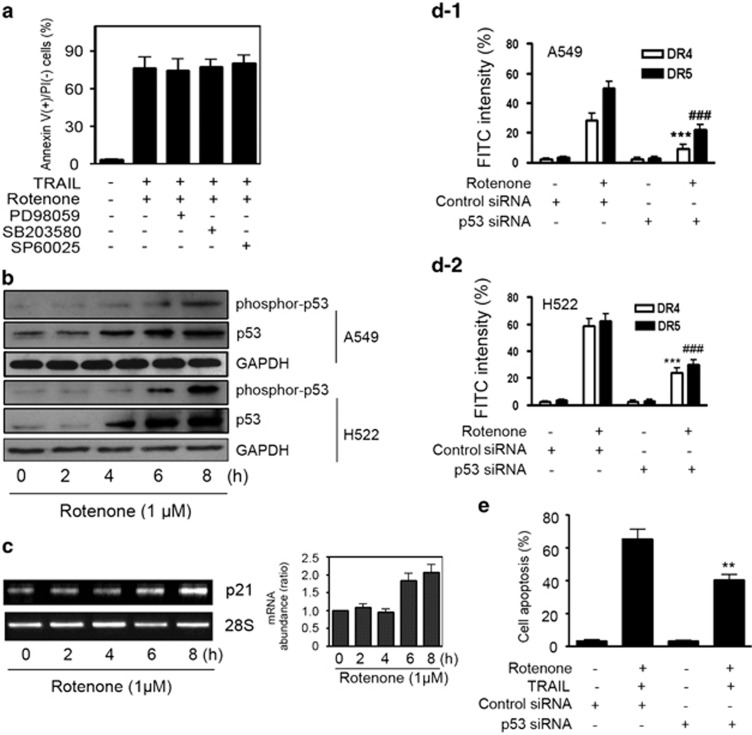
Involvement of p53 in the apoptosis-enhancing effect of rotenone. (**a**) A549 cells were pretreated with 20 *μ*M PD98059, SB203580 and SP60025 for 30 min, further treated with TRAIL (100 ng/ml) plus rotenone (1 *μ*M ) for an additional 8 h, after treatment, cell apoptosis was analyzed by annexin V/PI double staining method. (**b**) A549 or H522 cells were treated with rotenone at 1 *μ*M for 0, 2, 4, 6 and 8 h, respectively, after treatment, cell were collected and western blot analysis was performed to measure the protein expression levels of p53 as well as its phosphorylation. (**c**) A549 cells were treated with rotenone at 1 *μ*M for 0, 2, 4, 6 and 8 h, respectively. After treatment, RT-PCR was performed to measure p21 mRNA level. The quantitative result is shown on the right panel; the p21 mRNA expression in control cells was arbitrarily set at 1.0. (**d**) A549 cells in (**d-1**) or H522 cells in (**d-2**) were transfected with control or p53 siRNAs for 24 h, after transfection, cells were further treated with rotenone at 1 *μ*M for 8 h. After treatment, cells were collected and the surface expression levels of DR4 and DR5 on A549 and H522 cells were measured by flow cytometry. ***P<0.001, ^###^*P*<0.001, as compared with cells transfected with control siRNA. (**e**) A549 cells were transfected with control or p53 siRNAs for 24 h, after transfection, cells were further treated with TRAIL at 100 nM plus rotenone at 1 *μ*M for 8 h. After treatment, cells were collected and apoptosis was measured by annexin V/PI double staining method. **P<0.01, as compared with cells transfected with control siRNA

TRAIL receptors DR4 and DR5 are regulated at multiple levels. At transcriptional level, studies suggest that several transcriptional factors including NF-*κ*B, p53 and AP-1 are involved in DR4 or DR5 gene transcription.^2^ The NF-*κ*B or AP-1 transcriptional activity was further modulated by ERK1/2, JNK and p38 MAP kinase activity. Unexpectedly, we found here that none of these MAP kinases inhibitors were able to suppress the apoptosis mediated by TRAIL plus rotenone (Figure 3a). To find out other possible mechanisms, we observed that rotenone was able to stimulate p53 phosphorylation as well as p53 protein expression in A549 and H522 cells (Figure 3b). As a p53-inducible gene, p21 mRNA expression was also upregulated by rotenone treatment in a time-dependent manner (Figure 3c). To characterize the effect of p53, A549 cells were transfected with p53 siRNA. The results, as shown in Figure 3d-1 demonstrated that rotenone-mediated surface expression levels of DR4 and DR5 in A549 cells were largely attenuated by siRNA-mediated p53 expression silencing. Control siRNA, however, failed to reveal such effect. Similar results were also obtained in H522 cells (Figure 3d-2). Silencing of p53 expression in A549 cells also partially suppressed the apoptosis induced by TRAIL plus rotenone (Figure 3e).

### Rotenone suppresses c-FLIP expression and increases the sensitivity of A549 cells to TRAIL-induced apoptosis

**Figure 4:**
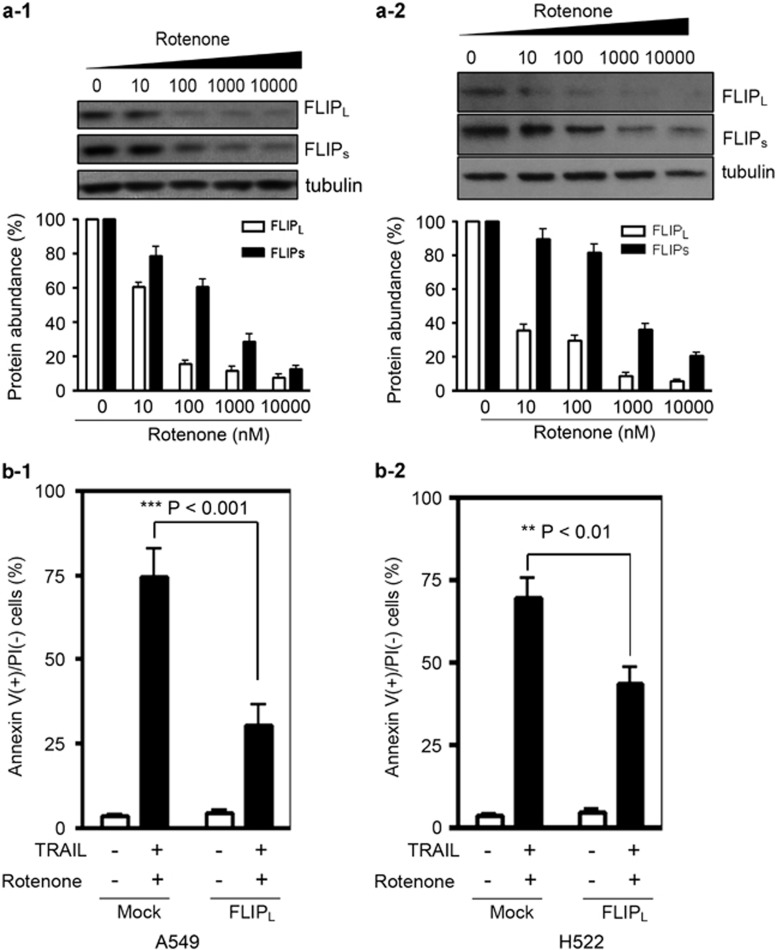
Effect of rotenone on c-FLIP expression in NSCLC cells. A549 cells (**a-1**) or in H522 cells in (**a-2**) were treated with rotenone at 0, 10, 100, 1000 and 10 000 nM, respectively, for 8 h. After treatment, cells were lyzed and subjected to western blot analysis. The quantitative result is shown below, and the protein abundance of c-FLIP_L_ and c-FLIP_s_ in control cells were arbitrarily set at 100%. A549 cells (**b-1**) or H522 cells in (**b-2**) were transfected with 2 *μ*g mock plasmid or c-FLIP_L_-overexpressing plasmid for 24 h, further treated with 100 ng TRAIL plus rotenone at 1 *μ*M for 8 h. After treatment, cells were collected and apoptosis was measured by annexin V/PI double staining. Each experiment was performed in triplicate. **P<0.01, ***P<0.001, as compared with mock plasmid transfected cells

The c-FLIP protein has been commonly appreciated as an anti-apoptotic molecule in death receptor-mediated cell apoptosis. In this study, rotenone treatment led to dose-dependent downregulation of c-FLIP expression, including c-FLIP_L_ and c-FLIP_s_ in A549 cells (Figure 4a-1), H522 cells (Figure 4a-2), H441 and Calu-1 cells (Supplementary Figure S4). To test whether c-FLIP is essential for the apoptosis enhancement, A549 cells were transfected with c-FLIP_L_-overexpressing plasmids. As shown in Figure 4b-1, the apoptosis of A549 cells after the combined treatment was significantly reduced when c-FLIP_L_ was overexpressed. Similar results were also obtained in H522 cells (Figure 4b-2).

### Bcl-X_L_ is involved in the apoptosis enhancement by rotenone

**Figure 5:**
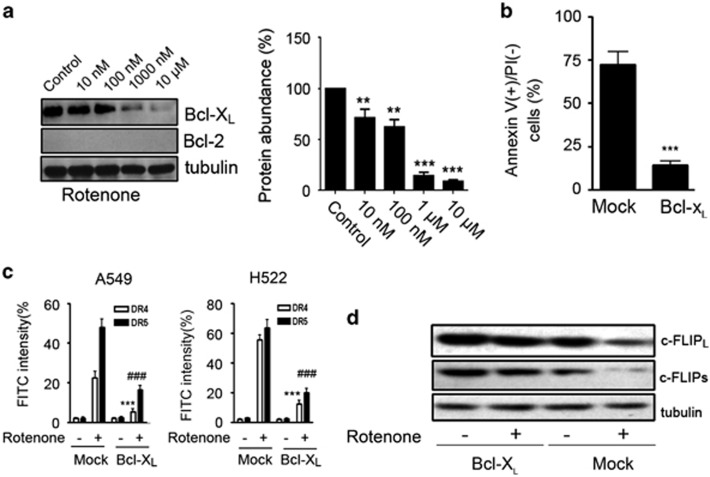
Involvement of Bcl-X_L_ in the apoptosis enhancement by rotenone. (**a**) A549 cells were treated with rotenone at 0, 10, 100, 1000 and 10 000 nM for 8 h, after treatment, western blot analysis was performed to measure Bcl-X_L_ and Bcl-2 expression levels, tubulin was included as a loading control. The quantitative analysis of Bcl-X_L_ protein expression in rotenone-treated A549 cells is shown on the right panel. The protein expression of Bcl-X_L_ in control cells was arbitrarily set at 100%. **P<0.01, ***P<0.001, as compared with control cells. (**b**) A549 cells were transfected with mock, or Bcl-X_L_-overexpressing plasmid for 24 h, then treated with TRAIL (100 ng/ml) plus rotenone (1 *μ*M ) for 8 h. Cell apoptosis was measured by annexin V/PI double staining. ***P<0.001, as compared with mock transfectant. (**c**) A549 or H522 cells were transfected with mock or Bcl-X_L_ plasmids for 24 h, after transfection, cells were treated with rotenone at 1 *μ*M for an additional 8 h, surface expression levels of DR4 and DR5 receptors were measured by flow cytometric analysis. ***P<0.001, ^###^*P*<0.001, as compared with mock transfectant. (**d**) A549 cells were transfected with mock or Bcl-X_L_ plasmids for 24 h, after transfection, cells were treated with rotenone at 1 *μ*M for an additional 8 h, western blot analysis was performed to examine the c-FLIP_L_ and c-FLIPs expression levels

Notably, c-FLIP downregulation by rotenone in NSCLC cells was irrelevant to p53 signaling (data not shown). To identify other mechanism involved, we found that anti-apoptotic molecule Bcl-X_L_ was also found to be downregulated by rotenone in a dose-dependent manner (Figure 5a). Notably, both Bcl-X_L_ and c-FLIP_L_ mRNA levels remained unchanged in cells after rotenone treatment (Supplementary Figure S5). Bcl-2 is homolog to Bcl-X_L_. But surprisingly, Bcl-2 expression was almost undetectable in A549 cells. To examine whether Bcl-X_L_ is involved, A549 cells were transfected with Bcl-X_L_-overexpressing plasmid. As compared with mock transfectant, cell apoptosis induced by TRAIL plus rotenone was markedly suppressed under the condition of Bcl-X_L_ overexpression (Figure 5b). To characterize the mechanisms, surface expression levels of DR4 and DR5 were examined. As shown in Figure 5c, the increased surface expression of DR4 and DR5 in A549 cells, or in H522 cells were greatly reduced after Bcl-X_L_ overexpression (Figure 5c). In addition, Bcl-X_L_ overexpression also significantly prevented the downregulation of c-FLIP_L_ and c-FLIP_s_ expression in A549 cells by rotenone treatment (Figure 5d).

### Rotenone suppresses the interaction between BCL-X_L_/p53 and increases PUMA transcription

**Figure 6:**
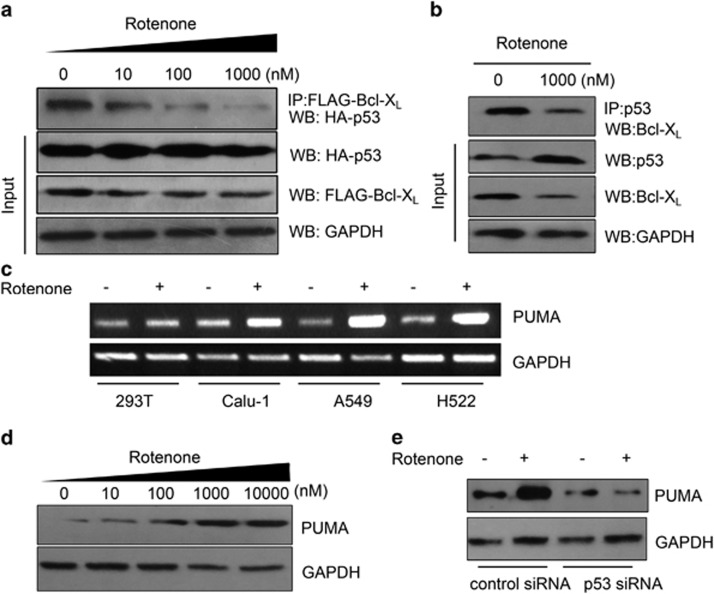
Effects of rotenone on Bcl-X_L_/p53 interaction and PUMA expression. (**a**) A549 cells were transfected with FLAG-tagged Bcl-X_L_ and HA-tagged p53 for 24 h, after transfection, cells were further treated with rotenone at 0, 10, 100 and 1000 nM for 8 h. After treatment, immunoprecipitation experiments were performed by using anti-FLAG IgG. The immunopellets were subject to western blot analysis by using anti-HA IgG. Cellular protein input of HA-tagged p53 and FLAG-tagged Bcl-X_L_ was also shown, GAPDH was included as a loading control. (**b**) A549 cells were treated with rotenone at 1 *μ*M for 8 h, after treatment, cells were lyzed and immunoprecipitation experiments were performed by using anti-p53 IgG to pull down p53 and the associated proteins. Cellular protein input of p53 and Bcl-X_L_ was also shown, GAPDH was included as a loading control. (**c**) The 293T, H522, A549 and Calu-1 cells were treated with rotenone at 1 *μ*M for 8 h, after treatment, cells were collected and RT-PCR analysis was performed to measure PUMA mRNA expression levels, GAPDH was included as a loading control. (**d**) A549 cells were treated with rotenone at 1, 10, 100, 1000 and 10 000 nM for 8 h, after treatment, western blot analysis was performed to examine the PUMA expression levels. (**e**) A549 were transfected with control or p53 siRNAs for 24 h, after transfection, cells were treated with rotenone at 1 *μ*M for an additional 8 h, western blot analysis was performed to examine the PUMA protein expression. The experiments were performed at least in triplicate

Lines of evidence suggest that Bcl-X_L_ has a strong binding affinity with p53, and can suppress p53-mediated tumor cell apoptosis.^26^ In this study, FLAG-tagged Bcl-X_L_ and HA-tagged p53 were co-transfected into cells; immunoprecipitation experiment was performed by using FLAG antibody to immunoprecipitate HA-tagged p53. As a result, we found that at the same amount of p53 protein input, rotenone treatment caused a concentration-dependent suppression of the protein interaction between Bcl-X_L_ and p53 (Figure 6a). Rotenone also significantly suppressed the interaction between endogenous Bcl-X_L_ and p53 when polyclonal antibody against p53 was used to immunoprecipitate cellular Bcl-X_L_ (Figure 6b). Recent study highlighted the importance of PUMA in BCL-X_L_/p53 interaction and cell apoptosis.^27^ We found here that rotenone significantly increased PUMA gene transcription (Figure 6c) and protein expression (Figure 6d) in NSCLC cells, but not in transformed 293T cell. Meanwhile, this effect was attenuated by silencing of p53 expression (Figure 6e).

### Mitochondria-derived ROS are responsible for the apoptosis-enhancing effect of rotenone

**Figure 7:**
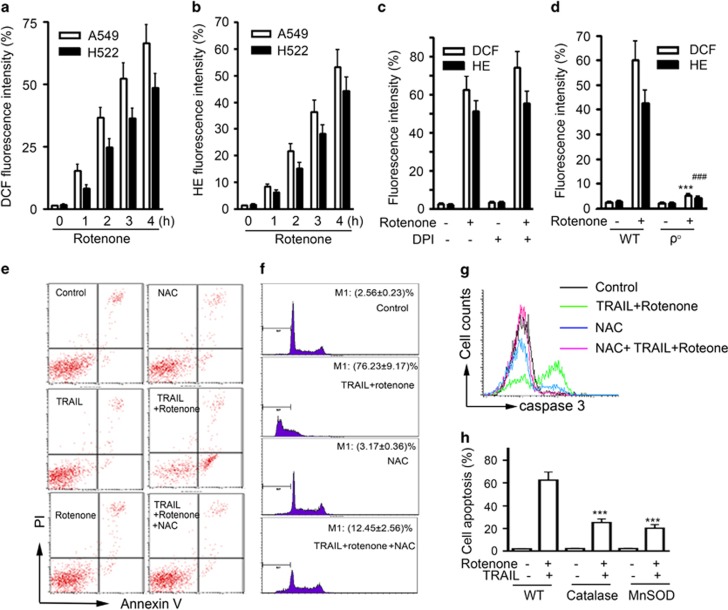
Involvement of ROS in rotenone-mediated apoptosis enhancement. (**a** and **b**) A549 or H522 cells were treated with rotenone at 1 *μ*M for 0, 1, 2, 3 and 4 h, respectively. After treatment, cells were collected and stained with DCFH for measurement of H_2_O_2_ generation in **a**, or with DHE for measurement of O_2_^.−^ in **b** by using flow cytometry. (**c**) A549 cells were pretreated with DPI at 10 *μ*M for 1 h, further treated with rotenone at 1 *μ*M for 4 h, after treatment, cells were stained with DCFH for measurement of H_2_O_2_, or with DHE for O_2_^.−^, respectively. (**d**) A549 WT or *ρ*° cells were treated with rotenone at 1 *μ*M for 4 h, after treatment, cells were stained with DCFH for measurement of H_2_O_2_, or with DHE for O_2_^.−^, respectively. ***P<0.001, ^###^*P*<0.001, as compared with wild-type A549 cells. (**e**–**g**) A549 cells were preincubated with NAC at 5 mM for 30 min, further treated with TRAIL (100 ng/ml) plus rotenone (1 *μ*M) for 8 h, cell apoptosis was measured by annexin V/PI double staining in (**e**), cell cycle analysis was performed by flow cytometry in (**f**), the caspase-3 cleavage activity was measured by flow cytometry by using FITC-conjugated substrate in (**g**). (**h**) A549 were transfected with mock, catalase or MnSOD-overexpressing plasmids for 24 h, after transfection, cells were treated with 100 ng TRAIL plus 1 *μ*M rotenone for an additional 8 h, cell apoptosis was measured by annexin V/PI double staining method. ***P<0.001, as compared with control cells

As an inhibitor of mitochondrial respiration, rotenone was found to induce reactive oxygen species (ROS) generation in a variety of transformed or non-transformed cells.^20, 22^ Consistently, by using 2',7'-dichlorofluorescin diacetate (DCFH) for the measurement of intracellular H_2_O_2_ and dihydroethidium (DHE) for O_2_^.−^, we found that rotenone significantly triggered the .generation of H_2_O_2_ (Figure 7a) and O_2_^.−^ (Figure 7b) in A549 and H522 cells. To identify the origin of ROS production, we first incubated cells with diphenylene iodonium (DPI), a potent inhibitor of plasma membrane NADP/NADPH oxidase. The results showed that DPI failed to suppress rotenone-induced ROS generation (Figure 7c). Then, we generated A549 cells deficient in mitochondria DNA by culturing cells in medium supplemented with ethidium bromide (EB). These mtDNA-deficient cells were subject to rotenone treatment, and the result showed that rotenone-induced ROS production were largely attenuated in A549 *ρ*° cells, but not wild-type A549 cells, suggesting ROS are mainly produced from mitochondria (Figure 7d). Notably, the sensitizing effect of rotenone on TRAIL-induced apoptosis in A549 cells was largely dependent on ROS, because the antioxidant *N*-acetylcysteine (NAC) treatment greatly suppressed the cell apoptosis, as shown in annexin V/PI double staining experiment (Figure 7e), cell cycle analysis (Figure 7f) and caspase-3 cleavage activity assay (Figure 7g). Finally, in A549 cells stably transfected with manganese superoxide (MnSOD) and catalase, apoptosis induced by TRAIL and rotenone was partially reversed (Figure 7h). All of these data suggest that mitochondria-derived ROS, including H_2_O_2_ and O_2_^.−^, are responsible for the apoptosis-enhancing effect of rotenone.

### Rotenone promotes BCl-X_L_ degradation and PUMA transcription in ROS-dependent manner

**Figure 8:**
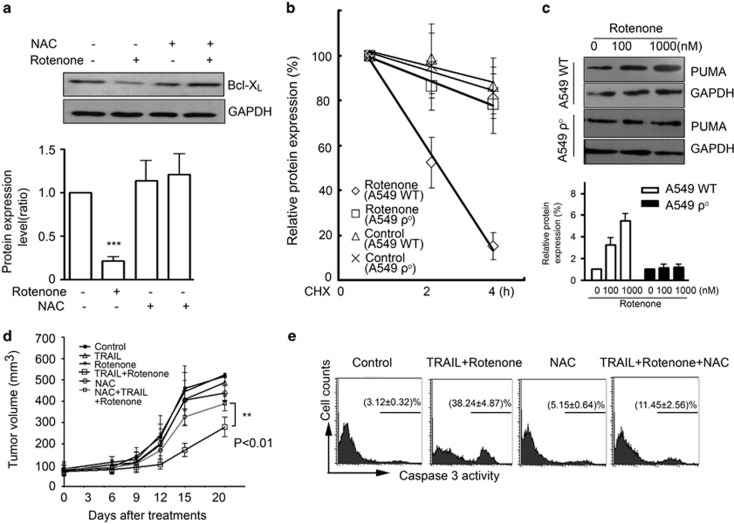
Involvement of ROS in rotenone-induced Bcl-X_L_ degradation and PUMA expression. (**a**) A549 cells were preincubated with NAC at 5 mM for 30 min, further treated with rotenone (1 *μ*M) for 8 h, western blot analysis was performed to examine Bcl-X_L_ expression levels. GAPDH was included as a control. The quantitative result of three independent experiments showing similar results was shown on the bottom panel. The Bcl-X_L_ expression in control cells was arbitrarily set at 1.0. ***P<0.001, as compared with control cells. (**b**) A549 WT or *ρ*° cells were treated with CHX at 5 *μ*M to halt protein synthesis, Bcl-X_L_ expression in cells after CHX treatment for 2 or 4 h was measured by western blot analysis. A549 WT or *ρ*° cells were also incubated in the absence or presence of rotenone at 1 *μ*M to examine the effect of rotenone on the degradation of Bcl-X_L_. (**c**) A549 WT or *ρ*° cells were treated with rotenone at 0, 100 and 1000 nM, respectively, after treatment, western blot analysis was performed to examine PUMA protein expression, GAPDH was included as a control. The quantitative result of western blot analysis is shown on the bottom panel. (**d**) A549 cells were inoculated into nude mice to produce xenografts model. Animals were challenged with 100 *μ*g TRAIL, 0.5 mg/kg rotenone or both once per 3 days for 21 consecutive days. For NAC treatment, animals were challenged with 600 mg/kg NAC once per 2 days. The tumor growth curve was plotted. **P<0.01, TRAIL+rotenone *versus* TRAIL+rotenone+NAC groups. (**e**) After experiment, the tumors were removed, and the caspase-3 activity in tumor cells was measured by flow cytometry by using FITC-conjugated caspase-3 substrate. The percentage of cells with activated caspase-3 activity in three independent experiments is indicated

To understand why ROS are responsible for the apoptosis-enhancing effect of rotenone, we found that rotenone-induced suppression of BCL-X_L_ expression can be largely reversed by NAC treatment (Figure 8a). To examine whether this effect of rotenone occurs at posttranslational level, we used cycloheximide (CHX) to halt protein synthesis, and found that the rapid degradation of Bcl-X_L_ by rotenone was largely attenuated in A549 *ρ*0 cells (Figure 8b). Similarly, rotenone-induced PUMA upregulation was also significantly abrogated in A549 *ρ*0 cells (Figure 8c). Finally, A549 cells were inoculated into nude mice to produce xenografts tumor model. In this model, the therapeutic effect of TRAIL combined with rotenone was evaluated. Notably, in order to circumvent the potential neurotoxic adverse effect of rotenone, mice were challenged with rotenone at a low concentration of 0.5 mg/kg. The results, as shown in Figure 8d revealed that while TRAIL or rotenone alone remained unaffected on A549 tumor growth, the combined therapy significantly slowed down the tumor growth. Interestingly, the tumor-suppressive effect of TRAIL plus rotenone was significantly attenuated by NAC (*P*<0.01). After experiment, tumors were removed and the caspase-3 activity in tumor cells was analyzed by flow cytometry. Consistently, the caspase-3 cleavage activities were significantly activated in A549 cells from animals challenged with TRAIL plus rotenone, meanwhile, this effect was attenuated by NAC (Figure 8e). The similar effect of rotenone also occurred in NCI-H441 xenografts tumor model (Supplementary Figure S6).

## Discussion

Restoration of cancer cells susceptibility to TRAIL-induced apoptosis is becoming a very useful strategy for cancer therapy.^28^ In this study, we provided evidence that rotenone increased the apoptosis sensitivity of NSCLC cells toward TRAIL by mechanisms involving ROS generation, p53 upregulation, Bcl-X_L_ and c-FLIP downregulation, and death receptors upregulation. Among them, mitochondria-derived ROS had a predominant role. Although rotenone is toxic to neuron, increasing evidence also demonstrated that it was beneficial for improving inflammation,^29^ reducing reperfusion injury,^30, 31^ decreasing virus infection^32^ or triggering cancer cell death. We identified here another important characteristic of rotenone as a tumor sensitizer in TRAIL-based cancer therapy, which widens the application potential of rotenone in disease therapy.

As Warburg proposed the cancer ‘respiration injury' theory, increasing evidence suggest that cancer cells may have mitochondrial dysfunction, which causes cancer cells, compared with the normal cells, are under increased generation of ROS.^33^ The increased ROS in cancer cells have a variety of biological effects.^34, 35, 36^ We found here that rotenone preferentially increased the apoptosis sensitivity of cancer cells toward TRAIL, further confirming the concept that although tumor cells have a high level of intracellular ROS, they are more sensitive than normal cells to agents that can cause further accumulation of ROS.^37^

Cancer cells stay in a stressful tumor microenvironment including hypoxia, low nutrient availability and immune infiltrates. These conditions, however, activate a range of stress response pathways to promote tumor survival and aggressiveness.^38^ In order to circumvent TRAIL-mediated apoptotic clearance, the expression levels of DR4 and DR5 in many types of cancer cells are nullified, but interestingly, they can be reactivated when cancer cells are challenged with small chemical molecules. Furthermore, those small molecules often take advantage of the stress signaling required for cancer cells survival to increase cancer cells sensitivity toward TRAIL. For example, the unfolded protein response (UPR) has an important role in cancer cells survival, SHetA2, as a small molecule, can induce UPR in NSCLC cell lines and augment TRAIL-induced apoptosis by upregulating DR5 expression in CHOP-dependent manner.^39^ Here, we found rotenone manipulated the oxidative stress signaling of NSCLC cells to increase their susceptibility to TRAIL. These facts suggest that cellular stress signaling not only offers opportunity for cancer cells to survive, but also renders cancer cells eligible for attack by small molecules. A possible explanation is that depending on the intensity of stress, cellular stress signaling can switch its role from prosurvival to death enhancement. As described in this study, although ROS generation in cancer cells is beneficial for survival, rotenone treatment further increased ROS production to a high level that surpasses the cell ability to eliminate them; as a result, ROS convert its role from survival to death.

Regulation of DR4 or DR5 expression is rather complex, mapping the promoter region of DR5 reveals multiple potential transcription factor binding sites including p53.^40^ DR5 was first cloned as a p53-responsive gene.^41^ In this study, we found rotenone-induced upregulation of DR5 was attenuated under the reduced expression of p53 by siRNA, suggesting that p53 may be an important trans-acting factor involved in DR5 transcription by rotenone. Notably, rotenone-induced DR4 upregulation was also mediated by p53, but no putative p53 binding sites are found on the sequence of DR4 promoter.^42^ We thus speculate that p53 may have an indirect stimulatory effect on DR4 transcription. Previous study suggests the sensitivity to TRAIL therapy was not closely associated with the expression levels of TRAIL receptors in some types of cancer.^25^ We found here that rotenone was able to increase DR4 and DR5 mRNA transcripts, protein expression and surface expression, furthermore, silencing of DR4 and DR5 expression in A549 cells led to significant suppression of cell apoptosis induced by TRAIL plus rotenone. All of these data suggest the upregulation of DR4 and DR5 receptors in NSCLC cells is important for the increased sensitivity by rotenone.

Both c-FLIP_L_ and c-FLIP_S_ are short-lived proteins and their expression levels are subject to regulation by ubiquitin/proteasome-mediated degradation.^43^ In general, c-FLIP expression is associated with chemoresistance. The downregulation of c-FLIP using antisense oligonucleotides^44^ or siRNA sensitizes cancer cells to chemotherapeutic agent-induced apoptosis, whereas overexpression of c-FLIP protects cells from apoptosis induced by certain cancer therapeutic agents such as etoposide and cisplatin.^45, 46, 47, 48^ In this study, rotenone decreased c-FLIP_L_ and c-FLIP_s_ expression levels in NSCLC, overexpression of c-FLIP_L_ partially suppressed the apoptosis by TRAIL plus rotenone, rotenone-induced downregulation of c-FLIP_L_ was abrogated by Bcl-X_L_ overexpression, all of these facts suggest that c-FLIP downregulation involves as an important mechanism for the apoptosis enhancement by rotenone. Interestingly, c-FLIP inhibits the extrinsic pathway of cell death, whereas Bcl-X_L_ inhibits the intrinsic pathway. Suppression of c-FLIP downregulation by Bcl-X_L_ suggests a possible crosstalk between intrinsic pathway and extrinsic pathway in rotenone-treated NSCLC. Whether Bcl-X_L_ regulates c-FLIP expression at transcriptional or posttranslational level needs more experiments to verify.

Reports have shown that cellular Bcl-X_L_ protein level is regulated by phosphorylation and ubiquitin-dependent proteasomal degradation,^49, 50^ or by deamidation process,^51^ or by apoptotic cleavage.^52^ Here, rotenone caused ROS-dependent Bcl-X_L_ degradation, raising a possibility that rotenone may induce the phosphorylation of Bcl-X_L_ and the subsequent Bcl-X_L_ ubiquitination and degradation, because previous study has demonstrated that ROS regulate phosphorylation and ubiquitination of Bcl-2 family proteins.^53^

The p53 is a well-known tumor suppressor, which has a key role in cellular stress response pathway. Bcl-2 family proteins are binding targets of p53, PUMA can release p53 from Bcl-X_L_/p53 complex and allow BAK and Bax to induce mitochondrial permeability.^26, 27, 54^ We found here rotenone stimulated ROS-dependent PUMA transcription, which in turn destroyed the Bcl-X_L_/p53 interaction. Both Bcl-X_L_ and p53 were involved in death receptors expression. We presume that the overexpressed Bcl-X_L_ may sequester p53 molecules and suppress its stimulatory effect on death receptors. As such, the effect of Bcl-X_L_ on death receptors expression may be indirect. As PUMA expression was regulated by p53, it seems reasonable to conclude here that under the treatment of rotenone, PUMA, Bcl-X_L_ and p53 form a positive feedback amplification loop to increase the apoptosis sensitivity. Mitochondria-derived ROS, however, promote the formation of this amplification loop.

MDM2 is a specific E3 ligase for p53 that can induce p53 ubiquitination and degradation.^55^ The strategy by disrupting the interaction between p53 and MDM2 has proven to be valuable for drug development.^56^ Following this fact, screening small molecules that can disrupt the interaction between p53/Bcl-X_L_ is also promising for drug development. We noticed here that the reduced interaction between p53 and Bcl-X_L_ by rotenone may be largely accounted by PUMA upregulation, however, the possibility that rotenone may have a direct inhibitory effect on the interaction between p53 and Bcl-X_L_ cannot be excluded.

In this study, it seemed that rotenone can sensitize both p53 wt (A549) and p53 null (Calu-1) NSCLC cells to TRAIL-mediated apoptosis. Moreover, rotenone increased the sensitivity of other p53 null NSCLC cells, including H526, H1437, H727, H441, H1299 and H510A to TRAIL (Supplementary Figure S7). As the majority of especially metastatic NSCLC cancers harbors p53 mutations, the therapeutic use of rotenone–TRAIL combination in p53 null NSCLC cancers may be of great significance.

In sum, the results of this study suggest that targeting mitochondrial respiration by rotenone is a promising approach to overcome the resistance of NSCLC cells to TRAIL treatment.

## Materials and Methods

### Reagents

Human recombinant TRAIL was cloned, and expressed in *Escherichia coli* BL21 (DE3) cells as previously described.^57^ Dulbecco's modified Eagle's medium (DMEM), lipofectamine 2000 and Trypan blue were from Invitrogen (Carlsbad, CA, USA). Rotenone, CHX and NAC were from Sigma (St. Louis, MO, USA). SP600125, PD98058 and SB203580 were from Calbiochem (San Diego, CA, USA). H_2_DCFDA and DHE fluorescent dyes were obtained from Molecular Probes (Eugene, OR, USA).

### Cell culture and preparation of human PBMCs

The human NSCLC cell lines including A549, H522, Calu-1 and H157 were purchased from American Type Culture Collection (ATCC, Manassas, VA, USA), and maintained in DMEM, which was supplemented with 10% heat-inactivated FBS, at 37 °C in a humidified incubator with 5% CO_2_. Human PBMCs were isolated by Ficoll/Paque (Pharmacia, Uppsala, Sweden) density gradient centrifugation of heparinized blood obtained from healthy adult donors. Cells were harvested from the inter-phase layer, washed in PBS twice and then resuspended in RPMI 1640 medium containing 10% (v/v) FBS, 10 mM glutamine and 50 U/ml penicillin/streptomycin. Cells were cultured in six-well tissue culture plates. Cell viability was assessed by Trypan blue.

### Plasmids

The expressing plasmids including FLAG-tagged Bcl-X_L_, HA-tagged p53 and c-FLIP_L_ were all generated in our lab by RT-PCR and subcloning method. Plasmid DNA was purified by plasmid DNA Maxi Purification Kit (Promega, Madison, WI, USA). The purified DNA was diluted to 1 mg/ml and stored at −20 °C until use. The plasmids were introduced into cells by PolyJet transfection reagent based on manufacturer's instruction.

### RNA interference

A549 cells were transfected with the siRNA for human TRAIL receptor DR4 and DR5,^58^ or for human p53.^29^ The transfection of siRNA was conducted in a 24-well plate using lipofectamine 2000. Forty-eight hours after the transfection, cells were treated with rotenone alone, TRAIL alone or their combination. Gene silencing effect was evaluated by western blot analysis and apoptosis was measured by annexin V/PI staining method.

### Semiquantitative RT-PCR

Total RNA was isolated using TRIZOL agent and chloroform extraction according to the seller's RNA extraction protocol. The PCR primers used to amplify DR4, DR5, PUMA, p21 and GAPDH were listed as follows: DR4: sense, 5′-TTGTGTCCACCAGGATCTCA-3′, antisense, 5′-GTCACTCCAGGGCGTACAAT-3′ DR5: sense, 5′-ACTCCTGGAATGACTACCTG-3′, antisense, 5′-ATCCCAAGTGAACTTGAGCC-3′ p21: sense, 5′-GGAACTTCGACTTTGTCACC-3′, antisense, 5′-AAGGCAGAAGATGTAGAGCG-3′ GAPDH: sense, 5′-CACCATCTTCCAGGAGCGAG-3′, antisense, 5′-GCAGGAGGCATTGCTGAT-3′ PUMA: sense, 5′-ACCTCAACGCACAGTACGAG-3′, antisense, 5′-GTATGCTACATGGTGCAGAG-3′.

### Western blot analysis

The immunoblot analysis was performed as previously described.^59^ Briefly, A549 cells after treatment were harvested and lysed, and the cleared lysate was separated by SDS-PAGE. After electrophoresis, proteins were transferred to PVDF membranes. The membranes were first hybridized with primary antibodies, and then with a horseradish peroxidase-conjugated anti-mouse, or anti-rabbit IgG secondary Ab (Sigma). FLAG, HA, GAPDH and tubulin antibodies were from Santa Cruz Biotechnology (Santa Cruz, CA, USA). Abs to p53, phosphor-p53, PUMA, c-FLIP_L_, c-FLIPs and Bcl-X_L_ were from Cell Signaling Technology (Beverly, MA, USA). The immune blots were developed using enhanced chemiluminescence system (Amersham Pharmacia Biotech, Amersham, UK).

### Measurement of caspase-3, -8 and -9 activities by flow cytometry

The caspase-3, -8 and -9 activities in NSCLC cells were determined by using CaspGLOW Green caspase-3, -8 and -9 staining kits (Biovision, Palo Alto, CA, USA) based on manufacturer's instructions.

### Measurement of surface DR4 and DR5 expression levels by flow cytometry

A549 cells (2 × 10^6^) before or after treatment were harvested and washed with ice-cold PBS. For measurement of antibody binding, cells were probed with primary antibody for 1 h. After incubation, cells were washed and resuspended in a 100 *μ*l binding buffer containing 2 *μ*l FITC-conjugated secondary IgG for another 1 h. Cells were then harvested, washed and resuspended in a 400 *μ*l ice-cold PBS and analyzed with flow cytometry (Becton Dickinson, Franklin Lakes, NJ, USA). Immediately before flow cytometric analysis, PI (1 *μ*g/ml) was added in each sample. Cells were divided into two population based on their permeability to PI, PI permeable or PI impermeable cells, which were individually gated to measure the mean FITC fluorescence in FL1 channel.

### Generation of stable cell lines transfected with human catalase or MnSOD cDNA

The full-length cDNA of human catalase and MnSOD were amplified by using RT-PCR reaction and cloned in frame into bicistronic pIRES-EGFP eukaryotic expression vector containing the gene encoding GFP. These constructs (pIRES-EGFP-catalase, pIRES-EGFP-MnSOD) were stably transfected into A549 cells by lipofectamine 2000. Transfected clones, derived from single colonies, were selected by limiting dilution in DMEM medium containing G418 (1.5 mg/ml) and screened for EGFP expression by flow cytometry, and further screened for catalase or MnSOD overexpression by western blot analysis. Control cell lines were generated by stably transfecting A549 cells with the pIRES-EGFP vector.

### Generation of A549 *ρ*° cells

A549 cell lacking mitochondrial DNA (*ρ*°) were generated by growing A549 cells in DMEM medium supplied with 10% fetal calf serum, 2 mM L-glutamine, 1 mM pyruvate, 50 *μ*g/ml uridine, 25 mM glucose and 50 ng/ml EB for 5–6 weeks.^60^ After selection, the cells were grown in the same medium without EB. Oxygen consumption was measured with a Clark-type oxygen electrode, and no oxygen uptake was observed for A549 *ρ*° cells.

### Measurement of intracellular H_2_O_2_ and O_2_^.^^−^

Generation of intracellular H_2_O_2_ was measured using H_2_DCFDA upon oxidation to the fluorescent derivative 2'–7'-dichlorofluorescin (DCF) by reactions with H_2_O_2_. Following treatment, cells were collected and resuspended in 500 *μ*l DMEM containing 2% FBS and 10 *μ*M H_2_DCFDA for 20 min at 37 °C. Subsequently, cells were washed with PBS and analyzed with flow cytometry (Becton Dickinson, Oxford, UK) with excitation set at 488 nm and emission at 530 nm. Immediately before flow cytometry analysis, PI (1 *μ*g/ml) was added to exclude the non-viable cells. For measurement of intracellular O_2_^.−^, cells after treatment were loaded with 5 *μ*M DHE, analyzed with flow cytometry in FL3 channel.^23^

### Assessment of cell apoptosis by annexin V and PI double staining

The annexin V/PI double staining method was performed based on previously described method.^59^ In brief, A549 cells after treatment were stained with EGFP-tagged annexin V (1 : 2000), PI was added immediately before analysis by flow cytometry. EGFP fluorescence emission was measured in the FL1 channel and PI fluorescence was measured in the FL3 channel after cell doublets were excluded by pulse processing. Five thousand cells were counted per sample. The data were analyzed with BD CellQuest software (Franklin Lakes, NJ, USA).

### Xenograft tumor models and *in vivo* delivery of drugs

A549 cells were grown in DMEM medium with 10% heat-inactivated FBS and 2 mM L-glutamine. Cells were suspended in calcium and magnesium-free PBS (pH 7.35–7.45), and implanted subcutaneously in the right flank of each mouse. The cell concentrations for implantation were 5 × 10^6^ cells/0.2 ml per mouse. Once tumor volume reached >50 mm^3^, animals were randomized so that all groups had similar starting mean tumor volumes. Tumor measurements were taken three times per week. Animals were individually monitored throughout the experiment. Tumor volume (mm^3^) was calculated using the ellipsoid formula: [D*(*d*^2^)]2, where ‘*D*' represents the largest diameter of the tumor, and ‘*d*' represents the smallest diameter. After establishment of A549 xenografts, animals were injected intraperitoneally with 100 *μ*g TRAIL, or rotenone at 0.5 mg/kg, or both once per 3 days for 21 consecutive days. For NAC treatment, animals were challenged with NAC at 600 mg/kg once per 2 days. At the end of the experiments, animals were killed and the tumors were removed, homogenized and stained with 1–2 *μ*l FITC-DEVD-FMK for detection of caspase-3 activities.

### Statistical analysis

The results were expressed as mean±S.E.M. The statistical analysis involving two groups was performed by means of Student's *t*-test. Analysis of variance followed by Dunnett's multiple comparison tests was used to compare more than two groups. All data were processed with SPSS 10.0 software (SPSS, Chicago, IL, USA).

## Abbreviations

c-FLIP: cellular FLICE-like inhibitory protein
DHE: dihydroethidium
DISC: death-inducing signaling complex
DPI: diphenylene iodonium
DR4/DR5: death receptor 4/5
EB: ethidium bromide
FADD: Fas-associated protein with death domain
MnSOD: manganese superoxide
NAC: *N*-acetylcysteine
NSCLC: non-small-cell lung carcinoma
PBMC: peripheral blood mononuclear cells
ROS: reactive oxygen species
TRAIL: tumor necrosis factor-related apoptosis-inducing ligand
UPR: unfolded protein response
