# Supplementary material for: Mitochondrial inhibitor sensitizes non-small-cell lung carcinoma cells to TRAIL-induced apoptosis by reactive oxygen species and Bcl-XL/p53-mediated amplification mechanisms
Source: Cell Death Dis. 2014 Dec 18;5(12):e1579–. doi: 10.1038/cddis.2014.547 (PMC4649849; doi:10.1038/cddis.2014.547)
Supplement: Supplementary Information [file cddis2014547x1.doc]

**Supplementary results**


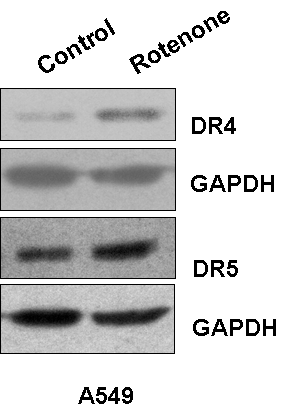


**Figure.S1. Effect of rotenone on death receptors expressions in A549 cells.**

A549 cells were treated with rotenone at 1 μM for 8 h, after treatments, the protein extracts of cells were examined by western blot analysis for measurement of DR4 and DR5 protein expressions. Three independent experiments were performed.


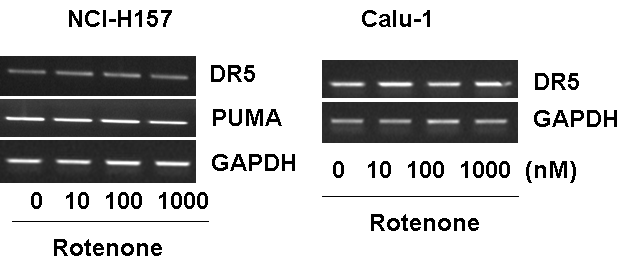


**Figure S2. Effect of rotenone on DR5 and PUMA mRNA expressions in p53 null NCI-H157 and Calu-1 cells.** Cells were treated with rotenone at 0, 10, 100, and 100 nM for 6 h, after treatments, the total RNA was extracted by TRIZOL. RT-PCR analysis was performed to examine DR5 and PUMA mRNA expressions. GAPDH was included as a control. Similar results were obtained in three independent experiments.


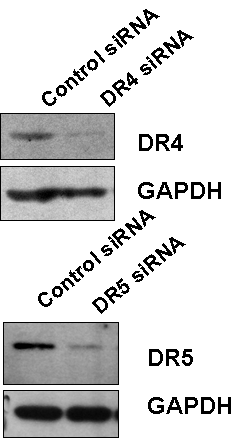


**Figure S3. Silencing of DR4 and DR5 expressions.** A549 cells were transfected with control siRNA, or siRNAs against DR4 and DR5 for 12 h, then challenged with rotenone at 1 μM for another 8 h, after treatments, cells were collected and the protein extracts of cells were examined by western blot analysis for measurement of DR4 and DR5 protein expressions. Three independent experiments were performed.


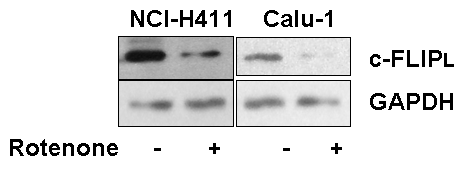


**Figure S4. Effect of rotenone on c-FLIP_L_ expression.** Both NCI-H411 and Calu-1 cells were treated with rotenone at 1 μM for 8 h, after treatments, the protein extract of cells were subject to western blot analysis for measurement of c-FLIP_L_ expression. Similar results were obtained in 3 independent experiments.


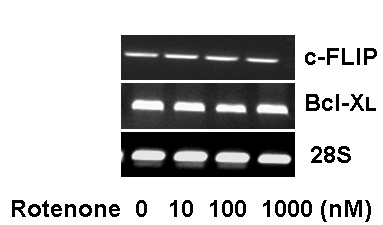


**Figure S5. Effect of rotenone on c-FLIP_L_ and Bcl-X_L_ mRNA expressions.**

A549 cells were treated with rotenone at 0, 10, 100, and 1000 nM for 8 h, after treatment, cells were collected and subject to RT-PCR analysis for measurement of c-FLIP_L_ and BCL-X_L_ mRNAs expression. 28S serves as a loading control.


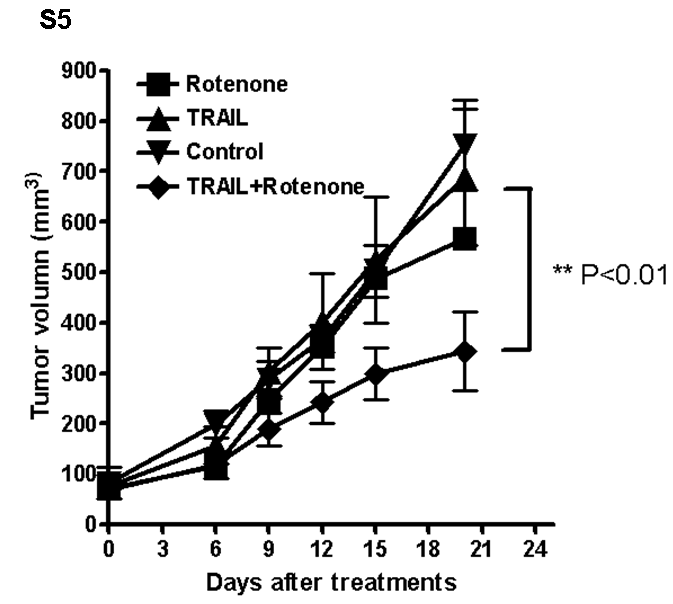


**Figure S6. Effect of rotenone in combination with TRAIL on H441 xenografts tumor model.** NCI-H441 cells were inoculated into nude mice to produce xenografts model. Animals were challenged with 100 μg TRAIL, 0.5mg/kg rotenone, or both once per three days for 21 consecutive days. The tumor growth curve was plotted. ** P<0.01, TRAIL vs. TRAIL+rotenone groups.


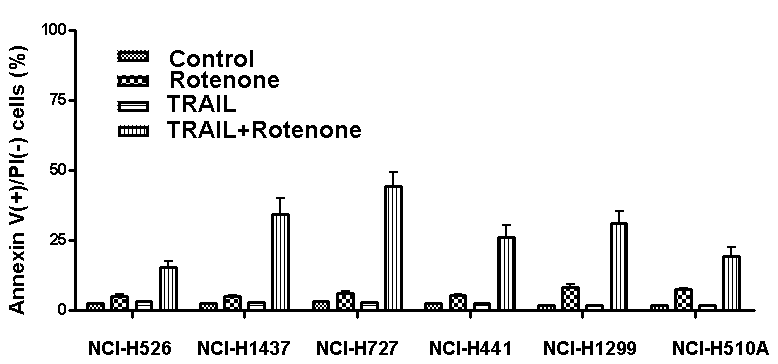


**Figure S7. Effects of rotenone, TRAIL, and TRAIL plus rotenone on p53 null NSCLC cells apoptosis.** NCI-H525, H1437, H727, H441, H1299, and H510A cells were treated with rotenone at 1 μM, TRAIL at 100 ng/ml, or both for 8 h, after treatments, cells were collected and the occurrence of apoptosis was measured by annexin V/PI double staining method. The experiment was performed in triplicate.
